# A Combination of Aqueous Extraction and Ultrafiltration for the Purification of Phycocyanin from *Arthrospira maxima*

**DOI:** 10.3390/microorganisms10020308

**Published:** 2022-01-28

**Authors:** Dante Matteo Nisticò, Amalia Piro, Daniela Oliva, Vincenzo Osso, Silvia Mazzuca, Francesco Antonio Fagà, Rosanna Morelli, Carmela Conidi, Alberto Figoli, Alfredo Cassano

**Affiliations:** 1 Laboratorio di Biologia e Proteomica Vegetale, Dipartimento di Chimica e Tecnologie Chimiche, Università della Calabria, Via P. Bucci 12/C, 87036 Rende, Italy; dante.nistico@unical.it (D.M.N.); amalia.piro@unical.it (A.P.); daniela.oliva@unical.it (D.O.); vincent0593@hotmail.it (V.O.); 2 BIORISI S.r.l.—Oil Fox Europe, Via G. Pinna 78, 88046 Lamezia Terme, Italy; direzione@biorisi.it; 3 Istituto per la Tecnologia delle Membrane (ITM-CNR), Università della Calabria, Via P. Bucci 17/C, 87036 Rende, Italy; r.morelli@itm.cnr.it (R.M.); c.conidi@itm.cnr.it (C.C.); a.figoli@itm.cnr.it (A.F.)

**Keywords:** *Spirulina*, phycocyanin, extraction, ultrafiltration, diafiltration

## Abstract

The purification of phycocyanin (PC) from *Spirulina* generally involves a combination of different techniques. Here, we report the results on PC yields from a combined aqueous extraction-ultrafiltration (UF) process of a strain of *Arthrospira maxima* cultivated in a farm devoted to producing PC with food-grade purity. Samples optimized from different biomass/solvent ratios were purified by using a polyethersulphone (PES) membrane with a molecular weight cut-off (MWCO) of 20 kDa. The UF system was operated at 2.0 ± 0.1 bar and at 24 ± 2 °C up to a volume concentration factor (VCF) of 5. A diafiltration (DF) process was conducted after UF in order to increase the PC recovery in the retentate. Samples were collected during both UF and DF processes in order to evaluate membrane productivity and PC purity. The average permeate fluxes of about 14.4 L/m^2^h were measured in the selected operating conditions and more than 96% of PC was rejected by the UF membrane independently ofthe extraction yields and times. The concentration of PC in the final retentate was 1.17 mg/mL; this confirmed the observed rejection and the final VCF of the process (about 5-fold when compared to the concentration of PC in the crude extract). In addition, the combination of UF and diafiltration allowed the removal of about 91.7% of the DNA from the crude extract, thereby improving the purity of the phycocyanin in the retentate fraction.

## 1. Introduction

Photosynthetic organisms, such as microalgae and cyanobacteriarepresent promising renewable sources of healthy food ingredients and functional food products due to their high contents of bioactive compounds, such as essential amino acids, antioxidant molecules, minerals and fibers. Compared to other natural sources of bioactive ingredients, these organisms have many advantages, including a wide biodiversity, the possibility to grow under conditions of low water utilization and the plasticity of their metabolism, which can be induced to produce specific molecules [1,2].

The production of microalgae-based protein products, for example, involve microalgae cultivation followed by harvesting, drying, cell disruption, protein extraction, hydrolysis and separation [3]. Among cyanobacteria, *Arthrospira platensis* (traditionally known as *Spirulina*), a blue-green coil shaped species, has received increased attention in receent years due to its high content of proteins, vitamins, minerals and many essential amino-acids and fatty acids [4]. It is an important source of phycocyanin (PC) and allophycocyanin (APC) water soluble proteins belonging to the phycobiliprotein family [5]. PC is a natural blue colorant, with an estimated molecular weight of 100–200 kDa; thanks to its therapeutic properties as an antioxidant, anti-inflammatory molecule, as well as its anti-cancer activities [6,7,8,9], the PC has great potential for industrial and commercial development. Its market value is estimated to be around 10–50 million US$ per annum [10]. 

The purification of PC from *Spirulina* and other microalgae has been investigated and optimized by several authors [11,12,13,14,15,16]. Purification generally involves different techniques that include extraction, centrifugation and separation by chromatography or ion exchange and dialysis. These procedures are time consuming and directly increase facilities and equipment-related expenses, thereby leading to an increase in production costs. In this context, membrane-based processes, such as microfiltration (MF) and ultrafiltration (UF), offer a useful approach as fractionation, purification and concentration steps asalternatives to expensive sequential purification techniques such as ammonium sulphate precipitation and gel filtration chromatography. These processes, thanks to their mild operating conditions, prevent possible thermal denaturation and deactivation of the PC molecules. Additional advantages over conventional technologies includean easy scale-up combined with high selectivity, modularity, low energy consumption, no phase change and no use of chemical additives [17,18]. 

UF membranes with a molecular weight cut-off (MWCO) of 50 kDa were used by Herrera et al. [19] to concentrate a spirulina extract up to a VCF of 1.9. A food grade phycocyanin powder with a purity ratio of 0.74 was obtained after adsorption on activated charcoal and spray drying. Jaouen et al. [20] investigated the use of MF and UF tubular inorganic membranes for the clarification of raw extracts after sonication of a spirulina culture, while UF, nanofiltration (NF) and reverse osmosis (RO) organic membranes were used for the concentration of the clarified extract. NF membranes exhibited the best performance in the concentration of the clarified extract: in selected conditions of operating pressure and tangential velocity (30 bar and 1.5 m/s, respectively), the recovery of PC and permeation flux resulted in 100% and 85 L/m^2^h, respectively.

A combination of an aqueous two-phase system, UF and precipitation was developed by Rito-Palomares et al. [15] in order to reduce the number of unit operations and increased the yield of the protein. In this approach, the use of a 30 kDa UF membrane followed by precipitation with ammonium sulfate led to a protein purity of 3.8 ± 0.1% and an overall product yield of 29.5% (*w*/*w*).

Figueira et al. [21] obtained a C-PC extract with purity of 0.95 suitable for use as a food dye (purity between 0.75 and 1.5) by using a combination of UF and 6 diafiltration cycles. In this approach, a 50 kDa polyethersulfone UF membrane in flat-sheet configuration was used.

Recently, Brião et al. [22] simplified the purification step by using the phosphate buffer extraction followed by UF and one-step of diafiltration (DF) membranes; throughthis procedure, a food-grade PC from *A. platensis* has been produced. Similarly, UF in diafiltration mode was assessed by Balti et al. [23] for protein fractionation of *A. platensis* in order to produce different protein extracts more or less purified, notably in salt and chlorophyll. 

This study aimed at developing a sustainable process for the recovery, fractionation and purification of PC from a strain of *A. maxima* extracts. It was based on a ‘green’ aqueous extraction of PC from the *Arthrosphira* biomass followed by a purification/fractionation step of the centrifuged extract through the use of a 20 kDa UF membrane aimed at removing mainly non-protein molecules, such as DNA, co-extracted with phycobiliproteins. To enhance the PC purification, UF was combined with a diafiltration (DF) step based on the addition of purified water to the UF retentate.

The effect of the biomass-water ratio on the PC concentration was investigated. Membrane productivity, cleaning efficiency and membrane retention towards PC and DNA were also assessed.

## 2. Materials and Methods

### 2.1. Chemicals

Bradford reagent, water CHROMASOLV^®^Plus for HPLC, bromophenol blue ACS reagent, ammonium bicarbonate BioUltra (≥99.5%) and acetonitrile anhydrous (99.8%) wereall acquired from Sigma-Aldrich (Milan, Italy). A 40% acrylamide/bis solution, ammonium persulfate, sodium dodecyl sulfate (SDS), trimethamine and glycine were purchased from Bio-Rad Laboratories (Hercules, CA, USA). Boric acid and ethylenediaminetetraacetic acid (EDTA) were acquired from Carlo Erba (Milan, Italy). Glycerol 99.0–101.0% was purchased from Honeywell Research Chemicals (Seelze, Germany). Coomassie R-250 solution was purchased fromAmersham Pharmacia Biotech (Uppsala, Sweden). Agarose CSL-AG5 for gel electrophoresis was purchased from Cleaver Scientific (Rugby, UK). Gel red nucleic acid gel stain 10,000× in water was purchased from Biotium (Fremont, CA, USA). Tetramethylethylendiamine (TEMED) was purchased from Chem-Lab (Zedelgem, Belgium). GeneRuler DNA Ladders 10Kpb was purchased from Thermo Fisher Scientific Inc. (Swindon, UK). All other reagents were of analytical grade.

### 2.2. Biological Materials

Dried biomass samples of the *Arthrospira maxima* strain cultivated according to the Oil Fox^®^ technology and registered trademark Spirulina-fox^®^ and covered by an international patent [24] were used.

### 2.3. Aqueous Extraction of Phycocyanin (PC) from A. maxima Biomass

The PC extraction from *A. maxima* samples was carried out at different biomass-solvent ratios in order to investigate the amount of solvent needed to maximize the yield of extraction. Ultrapure distilled water was added to dried *A. maxima* biomass to obtain suspensions of 200 mL at concentrations of 0.005, 0.01, 0.015 and 0.02 g/mL, respectively. All suspensions were stirred on a magnetic plate at 500 rpm for 24 h at RT. Three independent extractions were carried out and results of the PC concentration were expressed as mean value ± SD.

### 2.4. Feed Solution

After incubation time, the crude extract of *A. maxima* was centrifuged for 15 min at 6000 rpm, the supernatant removed and then frozen at −20 °C. The supernatant was used as a feed solution in the ultrafiltration experiments.

### 2.5. Ultrafiltration (UF): Experimental Set-Up and Procedure

Dead-end UF experiments were performed using the Sterlitech^TM^ HP 4750 high-pressure stirred cell (Sterlitech, Kent, WA, USA) with a filter area of 13.85 cm^2^ and a processing capacity of 300 mL. The cell filtration system was equipped with a flat-sheet polyethersulphone (PES) membrane (Nadir^®^ UP020 P, from Microdyn-Nadir GmbH, Wiesbaden, Germany) with a thickness of 210–250 μm and a nominal MWCO of 20 kDa. Filtration experiments were carried out according to a batch concentration configuration at a transmembrane pressure (TMP) of 2 bar and an operating temperature of 24 ± 2 °C. Stirring inside the cell was accomplished by using a magnetic stirrer. An initial volume of crude extract (UF feed) of 100 mL was used and the permeate was collected separately up to a final volume of 80 mL corresponding to a volume concentration factor (VCF) of 5. 

VCF is dimensionless and defined as:

(1) $$\mathrm{VCF}=\frac{V_{f}}{V_{r}}$$

where *V_f_* and *V_r_* are the feed and retentate volumes, respectively.

Diafiltration experiments were performed by adding distilled water to the UF retentate at the same flowrate of the permeate so as to keep the retentate volume constant (15 mL) during the process. Six diafiltration volumes (addition of 90 mL of distilled water) were added before stopping filtration. 

The diafiltration volume (ratio of the solvent volume added per volume of feed solution) was determined as follows [25]:

(2) $$DV=\frac{V_{p}}{V_{0}}=\frac{V_{w}}{V_{0}}$$

where *V_p_* and *V*_0_ are the volumes of permeate and feed solution, respectively, and *V_w_* is the volume of water added during the diafiltration process.

The diafiltration was operated at the same parameters of temperature, stirring conditions and transmembrane pressure asthe UF.

**Figure 1:**
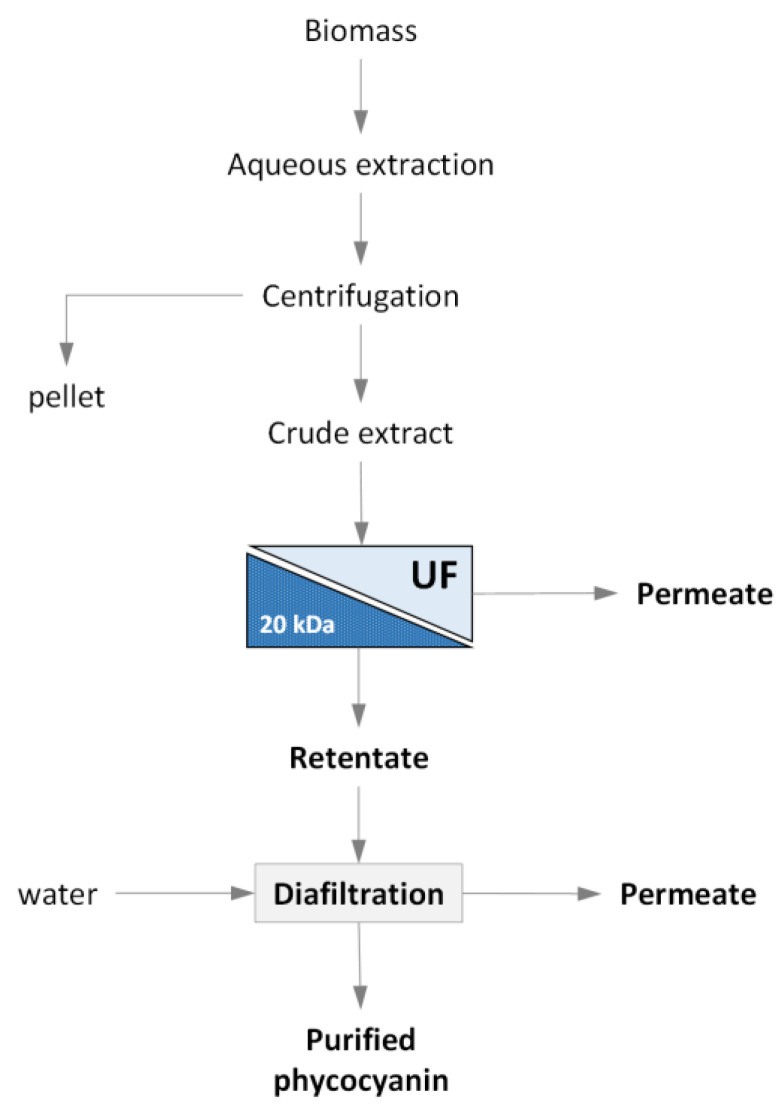
Schematic flowchart of the combined extraction/UF process investigated.

The schematic flowchart of the combined extraction–filtration process investigated in this study is depicted in Figure 1.

### 2.6. Data Processing

The permeate flux (*J_p_*), expressed in L/m^2^h, was determined by measuring the permeate volume collected in a given time according to Equation (3):

(3) $$J_{p}=\frac{V_{p}}{t\;A}$$

where *V_p_* (L) is the permeate volume, *t* (h) is the permeation time and *A* (m^2^) is the membrane area.

The hydraulic permeability of the UF membrane was determined by measuring the water permeate flux at different TMP values (at an operating temperature of 25 °C). The fouling index was determined by measuring the hydraulic permeability before and after filtration experiments, according to the following equation:

(4) $$FI=\left(\frac{W_{p1}}{W_{p0}}\right)\cdot 100$$

where *W_p_*_0_ and *W_p_*_1_ are the pure water permeability before and after UF experiments, respectively.

After the experiments with the crude extract, the UF membrane was cleaned in two steps. The first cleaning step was performed with distilled water for 30 min at a temperature of 40 °C in order to remove the reversible polarized layer. The hydraulic permeability measured afterwards was *W_p_*_2_. 

In the second step, the membrane was submitted to an enzymatic cleaning with a solution of Ultrasil P3 (Henkel-Ecolab, Dusseldorf, Germany) at 0.5% *w*/*w* for 30 min at 40 °C. At the end of the cleaning procedure, the membrane was rinsed with distilled water for 10 min and the hydraulic permeability, indicated as *W_p_*_3_, was measured.

The cleaning efficiency (*CE*) was evaluated by using the flux recovery method according to the following equation:

(5) $$CE=\left(\frac{W_{p3}}{W_{p0}}\right)\cdot 100$$

where *W_p_*_3_ is the water permeability measured after the enzymatic cleaning.

The rejection rate (*R*) of PC was calculated as described in Equation (6):

(6) $$R=\left(1-\frac{C_{p}}{C_{f}}\right)\cdot 100$$

where *C_p_* and *C_f_* refer to the concentration of PC within the permeate and feed, respectively.

### 2.7. Analytical Assays

#### 2.7.1. Phycocyanin (PC) Concentration

The PC concentration was measured by using a UV-VIS spectrophotometer (Shimadzu UV-160A, Kyoto, Japan) at wavelengths of 615 nm and 652 nm. The total amount of PC was calculated by using the following equation [26,27]:

(7) $$\mathrm{PC}=\frac{A_{615}-0.474\left(A_{652}\right)}{5.34}$$

where PC is the phycocyanin concentration (mg/mL), A_615_ is the optical density of the sample at 615 nm and A_652_ is the optical density of the sample at 652 nm. 

The PC purity was monitored spectrophotometrically by the A_615_/A_280_ ratio [28] according to the Equation (8):

(8) $$PP=\frac{A_{615}}{A_{280}}$$

where A_280_ is the optical density of the sample at 280 nm.

The purification factor (*PF*) was calculated from the ratio of PC concentration in purified (*PC_p_*) and crude extract (*PC_c_*) by Equation (9) [29]:

(9) $$PF=\frac{PC_{p}}{PC_{c}}$$

#### 2.7.2. Protein Analysis by SDS-PAGE of the Feed, Retentate and Permeate after UF

The protein concentration was measured by the Bradford method [30] and the protein sample was separated by the acrylammide gel electrophoresis under denaturant conditions (SDS-PAGE) [31]. The protein extract, solubilized in a loading buffer, was activated for 4 min at 100 °C. 20 µL for each sample were loaded onto a 12% polyacrylamide gel. The electrophoretic run was performed by placing the gel in the electrophoretic chamber filled with a buffer solution (Buffer Tris Glycine) and setting the amperage and voltage (60 mA in the stacking gel and 120 mA in the running gel at a power of 200 V). Once the run was over, the gels were placed in a Coomassie R-250 solution overnight and then destained by washing in water, ammonium bicarbonate and acetonitrile solution. The gels were scanned using the “GS800” (Biorad, Hercules, CA, USA) densitometer and analyzed with the “QuantityOne” software (Biorad, Hercules, CA, USA) to identify the polypeptide bands.

#### 2.7.3. DNA Detection

To evaluate the nucleic acid occurrence in the three fractions after ultrafiltration, the quantity of DNA molecules has been assessed by the NanoDrop™ One/One^C^ Microvolume UV-Vis Spectrophotometer (Thermo Fisher Scientific Inc., London, UK); absorbances at 260 nm and 280 nm were measured and concentrations were calculated as:

(10) $$\mathrm{DNA}\;\mathrm{concentration}\;\left(\mathrm{ng}/\mu L\right)={\;A}_{260\mathrm{nm}}\cdot \frac{50\;\mathrm{ng}/\mu L}{1a.u.}$$

The quality of the DNA has been evaluated by the direct measurements of the ratios A_260_/A_280_.

For the electrophoresis analysis, equal volumes of feed, permeate and retentate from the same UF assay were solubilized in an equal volume of loading buffer under stirring overnight at RT. Samples were separated in a 0.8% agarose gel using 0.5X TBE as the running buffer; bromophenol blue was added to the samples as a tracking dye. The 10 kbp DNA ladder was used (Thermo Fisher Scientific Inc.). After 30 min of running at 70 mA, 120 V, the gel image was digitalized using a UV Transilluminator (Consort bvba, Turnhout, Belgium) equipped with a photocamera.

All analytical measurements were performed in triplicate. Results were expressed as mean value ± SD.

## 3. Results

### 3.1. Aqueous Extraction of PC

**Figure 2:**
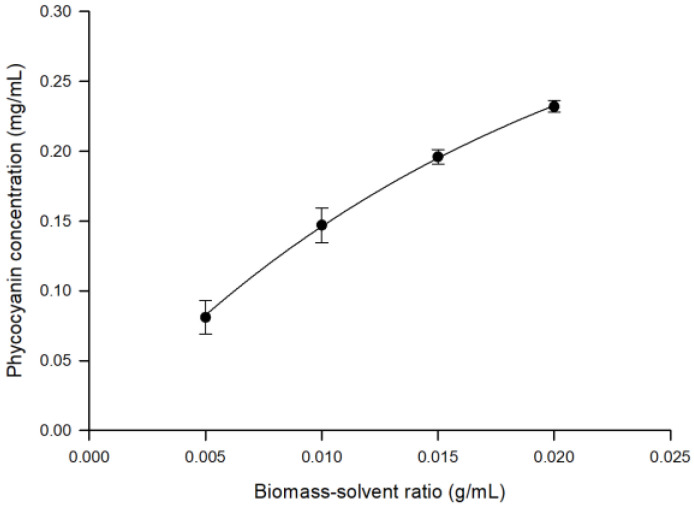
Extraction of phycocyanin from *A. maxima*. Effect of biomass-solvent ratio on phycocyanin concentration.

The variable biomass-solvent ratio strongly influences the PC concentration in the aqueous extract [32]. As illustrated in Figure 2, the maximum yield of PC (0.232 ± 0.0042 mg/mL) in the aqueous extract was obtained by using the largest biomass-solvent ratio (0.02 g/mL). Therefore, UF experiments were performed by using an aqueous extract sample obtained with a biomass-solvent ratio of 0.02 g/mL.

**Figure 3:**
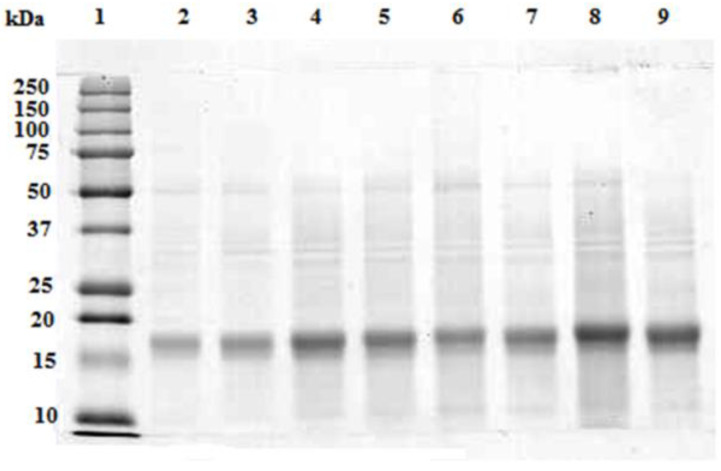
SDS-PAGE of the centrifuged extracts from 0.005 g/mL (lanes 2 and 3), 0.010 g/mL (lanes 4 and 5), 0.015 g/mL (lanes 6 and 7) and 0.020 g/mL (lanes 8 and 9) biomass-solvent ratio. 20 μL of each extract were loaded on the gel. Precision Plus ProteinTM Standards (Biorad, Hercules, CA, USA) were loaded on lane 1.

In Figure 3, the SDS-PAGE pattern of the supernatant from the crude extract at different biomass-solvent ratiosis reported. As it is observed, only a prominent polypeptide band at approx. 19 kDa was resolved in all aqueous extracts. The abundance of this band increased accordingly with the concentration of the extracts. Very weak bands at 50 kDa, 35 kDa and 10 kDa were also detected.The majority of A. maxima proteins, mainly the hydrophobic ones, remained in the biomass pellet, along with all the other water insoluble molecules (see the Supplementary Figure S1).

### 3.2. Ultrafiltration/Diafiltration

**Figure 4:**
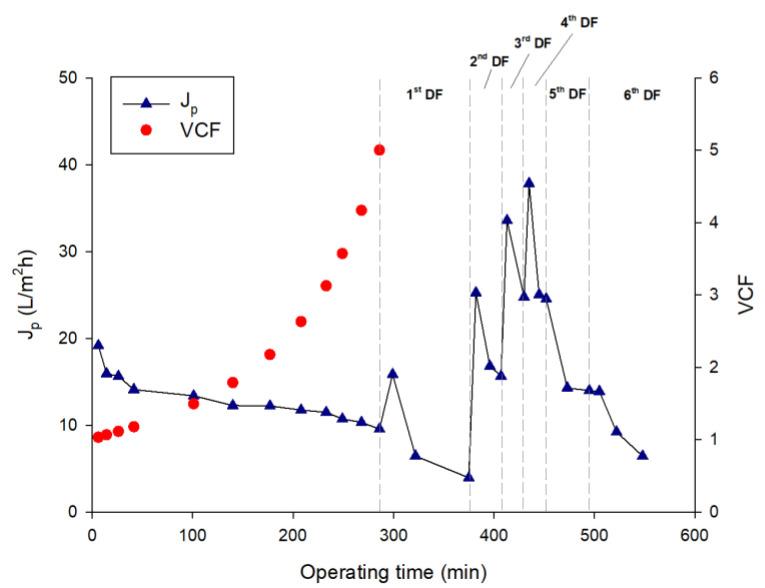
Ultrafiltration of *A. maxima* extract followed by six steps of diafiltration. Time course of permeate flux and volume concentration factor (VCF).

The feed solution was ultrafiltered with the selected membrane under operating conditions of pressure and temperature of 2 bar and 24 ± 2 °C, respectively. The initial permeate flux of about 19.2 L/m^2^h decreased sharply to 26.5% in the first steps of the process to reach a steady-state value of about 11.5 L/m^2^h at a VCF of 3.1; then, the permeate flux continued to decrease, reaching 9.5 L/m^2^h when the final VCF of 5 was reached (Figure 4). 

**Figure 5:**
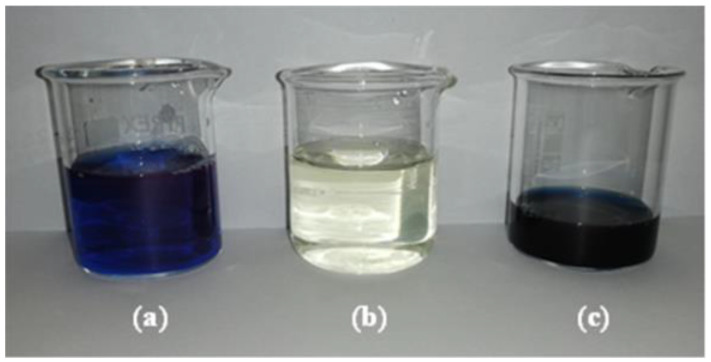
UF samples. (**a**) Aqueous crude extract (supernatant of centrifugation); (**b**) UF permeate; (**c**) UF retentate (at VCF 5).

**Figure 6:**
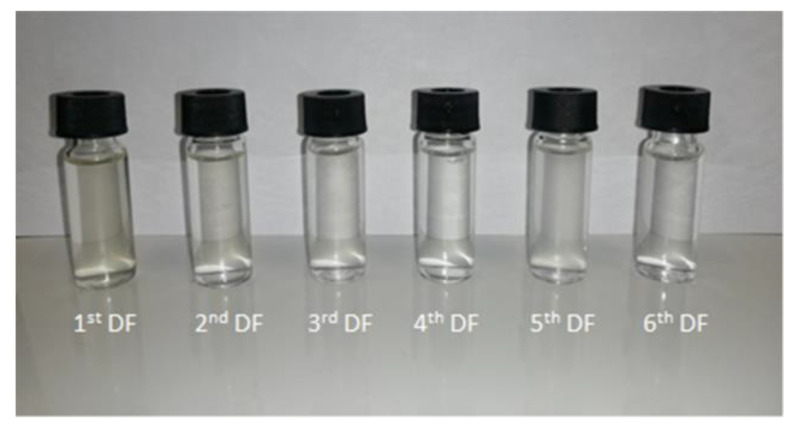
Permeate samples recovered in the course of diafiltration.

The addition of water in the DF steps produced a dilution of the retentate stream and an increase of the permeate flux at the beginning of each cycle of DF (with the exception of the last two DF steps) (Figure 4), followed by flux decay due to concentration polarization phenomena [22]. Figure 5 and Figure 6 illustrate the samples collected during the UF and DF steps, respectively.

**Figure 7:**
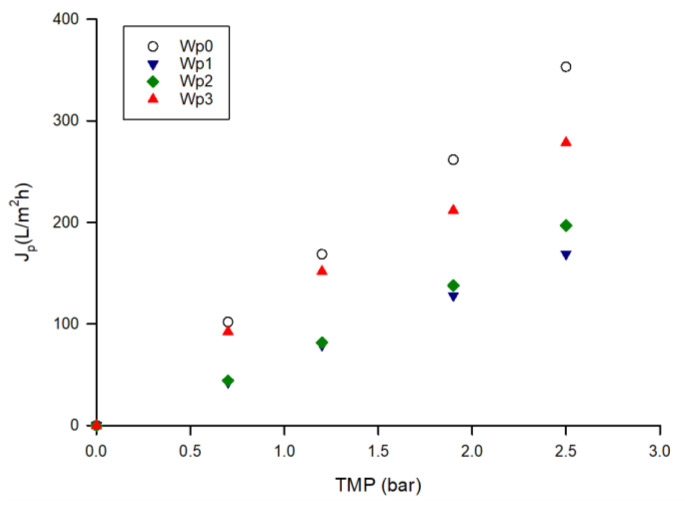
Water permeability of UF membrane before the treatment of aqueous extract and after cleaning procedures (*W*_*p*0_, initial water permeability; *W*_*p*1_, water permeability after treatment of aqueous extract; *W*_*p*2_, water permeability after cleaning with water; *W*_*p*3_, water permeability after enzymatic cleaning).

Figure 7 shows the pure water flux of the UF membrane before and after the treatment of the crude extract and after cleaning treatments. The initial water permeability of the membrane, of about 140.3 L/m^2^hbar, dropped to 67.0 L/m^2^hbar after the treatment of the crude extract. Therefore, the fouling index was estimated to be 52.3%. The cleaning with distilled water and enzymatic detergent at 40 °C allowed an increase in the membrane water permeability at 74.9 L/m^2^hbar and 114.2 L/m^2^hbar, respectively. Thus, the cleaning efficiency result was about 81%.

### 3.3. Analyses of Phycocyanin 

**Figure 8:**
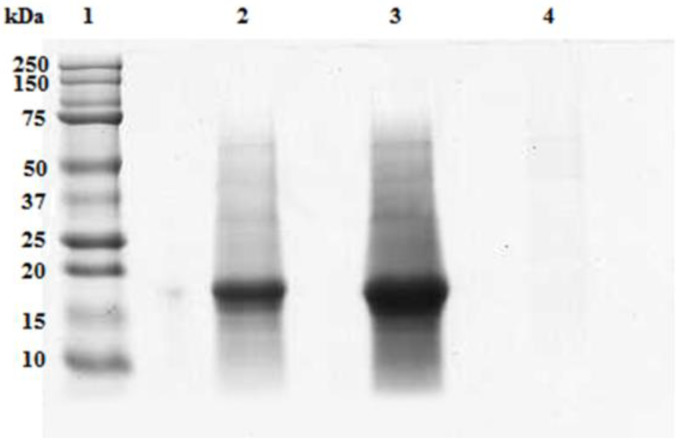
SDS-PAGE of the UF fractions from the aqueous extract of A. maxima at a concentration of 0.02 g/mL.An intense polypeptide band of approximately 19 kDa was resolved in the feed (lane 2) which became more intense in the retentate (lane 3) but was not detectable in the permeated fraction (lane 4). 20 µL of each fraction were loaded on the gel. Precision Plus ProteinTM Standards (Biorad, Hercules, CA, USA) wereloaded on lane 1.

In Figure 8, the electrophoresis of the fractions obtained after the UF treatment of the 0.020 g/mL feed solution is shown. Only one polypeptide band of aprox. 19 kDa was resolved in feed and retentates. This band appeared more concentrated in the retentate withrespect to the starting concentration of the feed fraction. No polypeptide band has been resolved in the permeate fraction.

**Figure 9:**
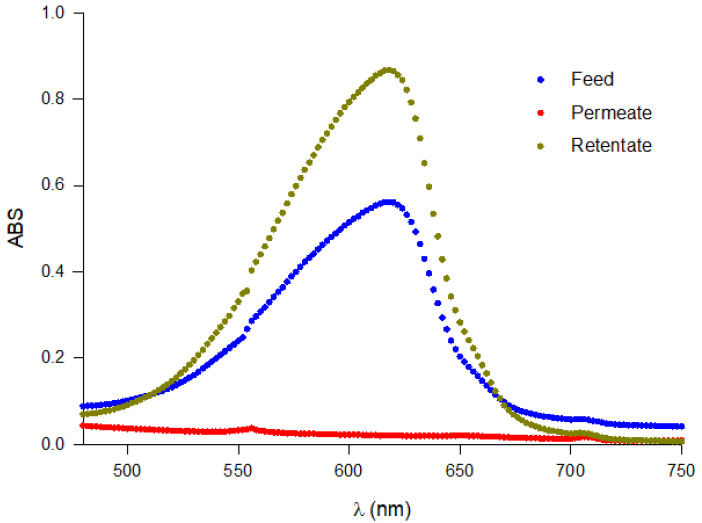
Full scan of the absorbance of UF fractions (feed, retentate and permeate).

In Figure 9, the absorbance spectra of the UF fractions in the range of 480–750 nm are reported. Higher absorbance was observed in the retentate fraction with respect to feed and permeate samples. The concentration of PC and related purity in the feed in the UF fractions and in the final retentate after diafiltration are reported in Table 1.

The concentration of PC in the retentate was4.8-fold higher than that of the feed solution and it was in agreement with the VCF of the process. The rejection of the UF membrane towards the PC was 96.2% and according to the mass balance of the process, 96.9% of the PC was recovered in the retentate.

The purity value of the crude extract increased from 0.74 to 0.93 in the UF retentate: accordingly, the UF process allowed a purification factor of 4.84. After six diafiltration cycles, the PC purity of the retentate and the purification factor increased to 1.16 and 5.06, respectively. This behavior can be attributed to the removal of most contaminant particles smaller than the nominal MWCO of the UF membrane (20 kDa) during the first 6 cycles of DF.

### 3.4. DNA Analyses

**Figure 10:**
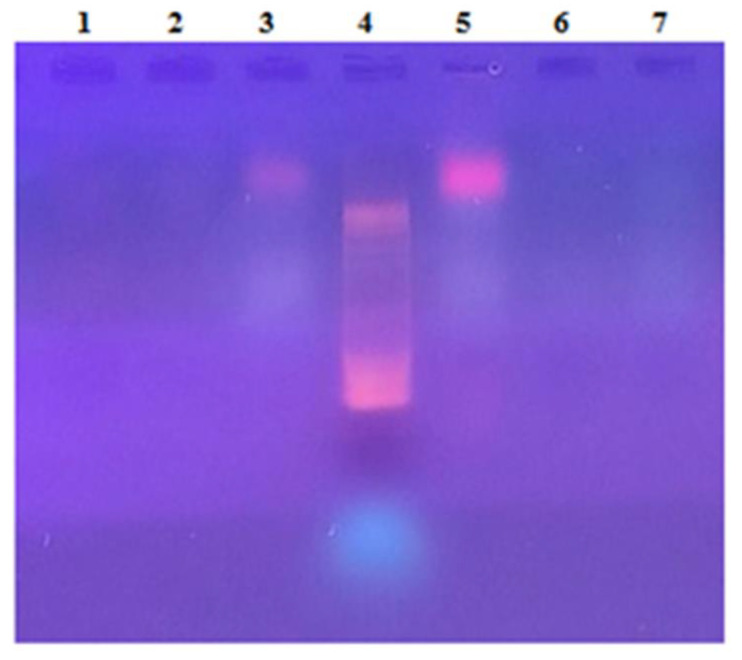
Agarose gel electrophoresis of the UF fractions obtained from the aqueous PC extracts from *A. maxima*. DNA bands were detected in the feeds (lanes 3 and 5) but not in the retentate fractions (lanes 1 and 6) nor in the permeate fractions (lanes 2 and 7). 10bp DNA ladder was used as electrophoresis markers (Lane 4).

The agarose gel electrophoresis of two independent extractions is reported in Figure 10. As can be seen, nucleic acid contamination occurred in the feed solution, suggesting that DNA molecules were co-extracted during solubilization with proteins from *A. maxima* biomass; after ultrafiltration, the DNA molecules were not detected in the retentate, suggesting that the DNA was efficiently removed; on the other hand, DNA was not detected in the permeate fraction, suggesting that during filtration DNA was degraded in very small fragments not detectable through electrophoresis in our conditions. This is corroborated by the evidence of the Abs 260/280 ratio values (Supplementary Table S1).

The contents of DNA measured in all fractions from the UF and diafiltration process of the feed solution are reported in Table 2.

According to the mass balance of the process, about 77% and 14% of DNA wasrecovered in the permeate and diafiltrate samples, respectively. Therefore, the combination of UF and DF allowed the removal of about 91.7% of the DNA from the crude extract, thereby improving the purity of the phycocyanin in the retentate fraction.

## 4. Discussion

The extraction protocol developed in this work obtained a very high phycocyanin yield from *A. maxima* biomass. The biomass-solvent ratio of 0.02 g/mL makes possible the use of smaller volumes of extractant in the purification steps and it agrees with data reported by Silveira et al. [29] in the optimization of PC extraction from *Spirulina platensis* using factorial design. The authors also reported that the use of a biomass-solvent ratio higher than 0.08 g/mL produced very concentrated suspensions so that the solvent was unable to promote an appropriate interaction with the biomass for efficient extraction.

It is well known that representative phycobiliproteins in *Spirulina* are phycocyanin and allophycocyanin [23,33]. It is interesting to point out that the allophycocyanin concentration measured in the original crude extract of *A. maxima* was about 0.01 mg/mL, 23-fold less than phycocyanin concentration. This unbalanced content of phycobiliproteins could mainly be attributed to our biological source of *A. maxima* cultivated according to the Oilfox^®^ technology [24].

The electrophoretic profile of *A. maxima* PC crude extract has shown that the majority of the water soluble proteins correspond to the molecular weight of approx. 19 kDa; in *Spirulina platensis,* the pure C-phycocyanin was reported to be formed by two subunits corresponding to α and β subunits of 17 and 21 kDa, respectively; as the molecular weight of the native purified C-PC was 115 kDa, the aggregation state of the purified C-PC was accomplished by the assemblage (αβ)3 [34]. Despite the literature on the C-phycocyanin extraction and purification from *A. maxima* [35,36,37], there is no information, to our knowledge, on the molecular characterization of C-phycocyanin in this species. Under denaturing conditions, our results suggest that the C-phycocyanin in *A. maxima* could consist of monomers with an apparent molecular weight of approximately 19 kDa. Another hypothesis is that, in our conditions, it is not possible to separate the two subunits that therefore appear merged into a single band. Further molecular studies are needed to clarify this finding.

The decrease in permeate flux observed during the UF of the crude extract in selected operating conditions could be attributed to pore blockage and cake formation mechanisms occurring sequentially during the filtration process. A larger drop in the permeate flux followed by long-term decay until reaching a steadystate of about 15 L/m^2^h was observed by Brião et al. [22] in the UF of a crude PC extract (0.53 mg/L) by using a PES UF membrane with a MWCO of 60 kDa. Higher fluxes (of the order of 50 L/m^2^h) were obtained by Chaiklahan et al. [38] by using membranes of 50 and 70 kDa; in this case, the crude extract was previously microfiltered in order to remove cellular fragments before UF.

The UF process has proven effective both in the purification of C-phycocyanin and its five-fold concentration in our experimental conditions regardless of the starting biomass-solvent ratio. Furthermore, UF combined with diafiltration solved the contamination by DNA.

The PC purity and the purification factor measured after the UF/DF treatment of the crude extract are in agreement with data reported by Figueira et al. [21] in the UF of a crude extract of *S. platensis* by using a 50 kDa PES membrane in a flat-sheet configuration. The authors reported a PC recovery between 60 and 99% and purification factors between 1.35 and 1.57 in different conditions of pressure (1, 1.5 and 2 kgf/cm^2^), pH (6, 6.5 and 7), temperature (4, 14.5 and 25 °C) and diafiltration cycles (0, 2 and 4). Similarly, Chaiklahan et al. [38] reported purity ratios of 1.11, 1.03 and 1.08 after the UF of the microfiltered extract with 50 kDa, 70 kDa and 100 kDa membranes, respectively. The purity ratio of the phycocyanin extract increased approximately 2-fold when compared to the initial purity (0.54).

On the other hand, Brião et al. [22] reported that the MF and UF membranes did not successfully purify the PC extracted from *Spirulina* by freeze-thaw extraction. Indeed, the PC purity of the crude extract decreased from 0.355 to 0.345 in the retentate of the MF process (with a flat-sheet polyvinylidene fluoride membrane of 0.4 μm as pore diameter) and from 0.403 to 0.382 in the retentate of the UF process (with a flat-sheet PES membrane with a MWCO of 60 kDa). These results were attributed to the formation of a thick cake layer which blocked the membrane pores, thus preventing the removal of lower molecular weight contaminants from the crude extract. The extraction with sodium phosphate buffer followed by UF (with a tubular PVDF membrane of 30–80 kDa) and one step of diafiltration allowed theenhancement of the purity of the crude extract up to 0.76, which in any case is lower than the result obtained in our work. In addition, the purification resulted in a 30% loss of antioxidant activity.

Literature data confirm that the combination of UF with diafiltration allow researchers to obtain C-phycocyanin extracts with purity between 0.75 and 1.5, which are suitable for use as food dye [21,38]. Higher purity ratios (up to >4) can be reached through the combination of UF with other technologies (i.e., ammonium sulfate precipitation, expanded-bed and fixed-bed ion exchange chromatography). Extracts suitable as cosmetic dye (purity of 2.1), biomarkers (purity of 3.0) and for therapeutic and biomedicine applications with an analytical grade (purity >4.0) were obtained by Figueira et al. [21] according to different combinations of processes, including UF.

After UF, electrophoresis showed that the band of C-phycocyanin is concentrated in the retentate fraction without changes in the molecular weight. Here, we can speculate that the native form of C-phycocyanin in the feed solution had the least dimeric conformation and therefore was retained by the membrane with a 20 kDa cut-off. Structural modifications might occur during C-phycocyanin ultrafiltration [21]; at the temperature of 25 °C, an increase in C-phycocyanin recovery was reported. This increase can be attributed to higher retention due to a slight increase in the molar mass of C-phycocyanin.

## 5. Conclusions

A green and sustainable method for the extraction and purification of phycocyanin (PC) from a strain of *Arthrospira maxima* has been developed. Aqueous extracts optimized from different biomass-solvent ratios were purified by ultrafiltration (UF) by using a 20 kDa membrane in a flat-sheet configuration. The UF process allowed the recovery of more than 96% of the PC in the retentate fraction and the removal of about 91.7% of the DNA content from the crude extract. The purity value of the crude extract increased from 0.74 to 0.93 after the UF process. The application of 6 DF cycles enhanced the purification process, thereby obtaining a C-PC extract with a purity degree of 1.16, which is suitable for human food use. The combination of UF with other methodologies (i.e., ammonium sulfate precipitation and ion exchange chromatography) could be a useful approach forenhancing the purity degree of DNA-free ultrafiltered samples, thereby allowing the production of extracts suitable for other uses including therapeutic and biomedicine applications.

## Figures and Tables

**Table 1:** Concentration and purity of PC obtained by aqueous extraction followed by UF and diafiltration (PF, purification factor).

Samples	PC Concentration (mg/mL)	PC Purity	PF
Feed	0.232 ± 0.0042	0.74 ± 0.030	-
Permeate	0.0089 ± 0.00085	-	-
Retentate	1.124 ± 0.0234	0.93 ± 0.009	4.84
Final retentate	1.174 ± 0.0244	1.16 ± 0.010	5.06

**Table 2:** Mass balance of DNA in both UF and DF processes.

Sample	DNA (ng/µL)	Volume	DNA (µg)
Feed	425 ± 8.5	100	42,500.0 ± 637.5
UF Permeate	413 ± 8.2	80	33,040.0 ± 495.6
UF Retentate	473 ± 9.4	20	9460.0 ± 141.9
DF1	181 ± 3.6	15	2715.0 ± 40.7
DF2	107 ± 2.1	15	1605.0 ± 24.1
DF3	52 ± 1.0	15	780.0 ± 11.7
DF4	25 ± 0.5	15	375.0 ± 5.6
DF5	20.5 ± 0.4	15	307.5 ± 4.6
DF6	11 ± 0.2	15	165.0 ± 2.4
DF tot			5947.5 ± 89.2
Permeate			33,040.0 ± 495.6
DFtot + Permeate			38,987.5 ± 584.8

## Data Availability

Not applicable.

## Supplementary Materials

The following supporting information can be downloaded at:https://www.mdpi.com/article/10.3390/microorganisms10020308/s1, Figure S1: SDS-PAGE of the pellet after the centrifugation of the extract from 0.005 g/mL (lanes 1), 0.010 g/mL (lanes 2), 0.015 g/mL (lane 4) and 0.020 g/mL (lane 5) biomass-solvent ratio. 20 µL of each extract wasloaded on the gel. Precision Plus ProteinTM Standards (Biorad, Hercules, CA, USA) was loaded on the lane 3; Table S1: Values of DNA absorbance at 260 and 280 nm in the aqueous PC solution (feed), UF and DF fractions and the respective absorbance ratios.
