# Supplementary material for: A Combination of Aqueous Extraction and Ultrafiltration for the Purification of Phycocyanin from Arthrospira maxima
Source: Microorganisms. 2022 Jan 28;10(2):308. doi: 10.3390/microorganisms10020308 (PMC8880360; doi:10.3390/microorganisms10020308)
Supplement: Supplementary file 1 [file microorganisms-10-00308-s001.zip › microorganisms-1534033-supplementary.pdf]

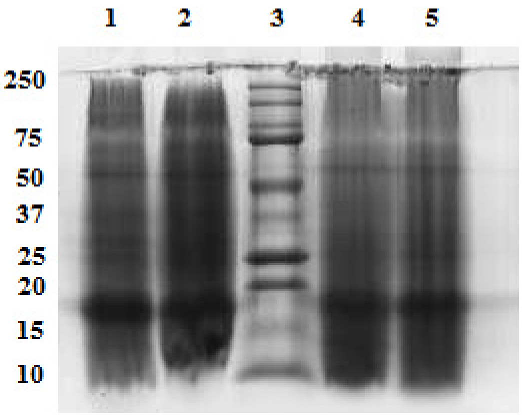

Figure S1. SDS-PAGE of the pellet after the centrifugation of the extracts from 0.005 g/mL (lanes 1), 0.010 g/mL (lanes 2), 0.015 g/mL (lane 4) and 0.020 g/mL (lane 5) biomass-solvent ratio. 20 L of each extracts were loaded on the gel. Precision Plus ProteinTM Standards (Biorad, Hercules, California, USA) was loaded on the lane 3

Table S1. Values of DNA absorbance at 260 and 280 nm in the aqueous PC solution (feed), UF and DF fractions and the respective absorbance ratios.

**Sample**	**DNA** (ng/µl)	**Abs 260 nm**	**Abs 280 nm**	**260/280 ratio**
Feed (0.020 g/mL)	425.0 ± 8.5	8.376 ± 0.126	5.25 ± 0.08	1.59 ± 0.02
Permeate	413.0 ± 8.2	6.278 ± 0.094	3.46 ± 0.05	1.81 ± 0.02
DF1	181.0 ± 3.6	3.618 ± 0.054	2.15 ± 0.03	1.68 ± 0.02
DF2	107.0 ± 2.1	2.141 ± 0.032	1.30 ± 0.02	1.64 ± 0.02
DF3	52.0 ± 1.0	1.032 ± 0.015	0.65 ± 0.01	1.58 ± 0.02
DF4	25.0 ± 0.5	0.501 ± 0.008	0.34 ± 0.01	1.49 ± 0.02
DF5	20.5 ± 0.4	0.411 ± 0.006	0.34 ± 0.01	1.2 ± 0.01
DF6	11.0 ± 0.2	0.218 ± 0.003	0.21 ± 0.00	1.02 ± 0.01
